# Use of gastrointestinal syndromic multiplex molecular assays and detection of *Escherichia coli* pathotypes in pediatric wards

**DOI:** 10.1128/jcm.01073-24

**Published:** 2025-02-26

**Authors:** Etienne Bizot, Stéphane Bonacorsi, Pauline Labé, Yael Pinhas, Aurélie Cointe, Agnès Ferroni, Jérémie F. Cohen, Hervé Lécuyer, Julie Toubiana

**Affiliations:** 1 Department of General Pediatrics and Pediatric Infectious Diseases, Necker-Enfants Malades, University Hospital, Assistance Publique - Hôpitaux de Paris (AP-HP), Université Paris Cité 555089 , Paris, France; 2 Microbiology Unit, Robert Debré Hospital, Assistance Publique - Hôpitaux de Paris 26930 , Paris, Île-de-France, France; 3 IAME, UMR1137, INSERM, Université Paris Cité 555089 , Paris, France; 4 Department of Clinical Microbiology, Necker-Enfants Malades University Hospital, Assistance Publique - Hôpitaux de Paris (AP-HP), Université Paris Cité 555089 , Paris, France; 5 INSERM U1151, CNRS UMR8253, Université Paris Cité, Institut Necker Enfants Malades, Paris, France; 6 Biodiversity and Epidemiology of Bacterial Pathogens, Institut Pasteur, Université Paris Cité 27058 , Paris, France; National Institute of Allergy and Infectious Diseases, Bethesda, Maryland, USA

**Keywords:** pediatric diarrhea, gastrointestinal PCR, multiplex PCR, *E. coli*, syndromic PCR

## Abstract

*Escherichia coli* pathotypes are enteric pathogens detected in gastrointestinal multiplex polymerase chain reaction (mPCR), with controversial clinical relevance. Our study aimed to describe clinical features and therapeutic decisions associated with *E. coli* detections in gastrointestinal mPCR. Children with positive mPCR for enteroaggregative (EAEC)*,* enteropathogenic (EPEC)*,* enterotoxigenic (ETEC), Shiga toxin-producing *E. coli* (STEC), and enteroinvasive *E. coli* (EIEC)/*Shigella* identified in two pediatric hospitals over 18 months (2020–2021) were included. We described the frequency of *E. coli* detection and subsequent modifications in antibiotic strategies. Among the 2,471 mPCRs performed, 338 (14%) tested positive for at least one *E. coli* pathotype. The patient's mean age was 4.2 years, with 95% experiencing gastrointestinal symptoms. Clinical presentation was generally comparable between *E. coli* pathotypes. A recent travel abroad was reported in 68/338 (20%) cases and was mainly observed in EIEC/*Shigella* infections. An *E. coli* was detected alone in 177/338 (52%) cases and with another virus, bacteria, or parasite in 161 (48%) cases. Multiple enteric pathogens were mainly detected with ETEC (*n* = 24/26, 92%) and EAEC (*n* = 82/121, 68%) detections. Antibiotic therapy was prescribed in 136/338 (40%) cases, with initiation based on mPCR results in 69/338 (20%). No antibiotic therapy was discontinued following positive mPCR results. Among the 69 initiations, 31 were deemed inappropriate after retrospective chart review. *E. coli* detection with mPCR tests may lead to inappropriate antibiotic initiation. Caution is advised when interpreting results from gastrointestinal mPCRs in children, as clinicians may be unaware of their often unclear or irrelevant clinical significance.

**IMPORTANCE:**

*Escherichia coli* pathotypes are increasingly detected through the widely used syndromic gastrointestinal multiplex PCR panels. However, their clinical significance and impact on antibiotic therapy in children remain uncertain. This study describes the clinical and microbiological characteristics associated with *E. coli* detections, as well as the subsequent modifications in antibiotic strategies. It highlights the frequent detection of *E. coli* pathotypes, often in association with other enteric pathogens, and reveals that nearly half of the antibiotics prescribed following these results were deemed inappropriate. These results underscore the need to enhance clinicians' interpretation of *E. coli*-positive results and reassess treatment strategies to optimize patient care.

## INTRODUCTION

Infectious diarrhea is one of the most frequent pediatric illnesses (1). The most common cause of acute diarrhea is a viral infection (1). However, patients with bacterial diarrhea may exhibit similar clinical symptoms. Several studies have described a significant rate of co-infections, such as viral-viral or viral-bacterial infections in children with diarrhea (2–4). Detecting the pathogens involved in pediatric diarrhea might sometimes be challenging with standard procedures, as microbial detection in stool is routinely based on standard culture for bacteria, while viruses are usually detected with antigen-based diagnostic tests or specific polymerase chain reaction (PCR) assays. Recently, syndromic multiplex PCR (mPCR) panels have been developed to detect simultaneously various bacteria, viruses, and parasites in stool specimens (5). These technologies offer several advantages: faster results (within a few hours, compared to a 1–2-day delay for standard stool culture), reduced technical time, increased sensitivity (6, 7), especially for *Campylobacter* that may yield negative in culture (8), and simultaneous detection of up to 24 pathogens.

Previous studies have shown that a gastrointestinal mPCR (FilmArray BioFire, BioMerieux) could detect at least one pathogen in 70–98% of children with bloody diarrhea or traveler's diarrhea (3, 4). All available commercial gastrointestinal mPCRs can detect several bacteria that are not isolated in standard stool culture, such as enteroaggregative *E. coli* (EAEC), enteropathogenic *E. coli* (EPEC), enteroinvasive *E. coli* (EIEC), enterotoxigenic *E. coli* (ETEC), or Shiga toxin-producing *Escherichia coli* (STEC) (5). This high level of detection of gastrointestinal pathogens may provide diagnostic keys to distinguish between inflammatory and infectious colitis in cases with persistent diarrhea, potentially reducing unnecessary procedures such as endoscopy and abdominal imaging (7). It also might improve our knowledge about the microbial epidemiology of infectious diarrhea. However, because of their high sensitivity, mPCRs may yield positive results of unclear or irrelevant clinical significance, with subsequent negative impact on antimicrobial and diagnostic stewardship efforts.

The impact of these new mPCRs on treatment decisions in children has been poorly studied. A previous French study found that antibiotic therapy was initiated in 16.3% of cases after a positive mPCR result, and only 0.6% was discontinued (4). Because of all the potential benefits mentioned above (3), mPCRs are increasingly being introduced into routine hospital care. However, the clinical relevance—or lack thereof—of *E. coli* pathotypes is not well-known among clinicians nor is the presence of healthy carriage of these pathotypes. Consequently, the detection of *E. coli* could lead to inappropriate antibiotic treatment. In our study, we evaluated the frequency of *E. coli* detection in gastrointestinal mPCRs and described clinical and microbiological characteristics as well as therapeutic decisions associated with these infections.

## MATERIALS AND METHODS

### Setting and participants

We conducted a multicentric retrospective study in two public university tertiary hospitals: Necker-Enfants malades (Center 71,000 inpatient admissions and 82,000 emergency room visits per year) and Robert-Debré (Center 46,000 inpatient admissions and 98,000 emergency room visits per year) hospitals, Assistance Publique - Hôpitaux de Paris in Paris, France, between 01 June 2020 and 31 December 2021. These two centers offer a wide range of medical pediatric specialties, surgical specialties, and intensive care units. The study was started according to the beginning of (i) computerized results for mPCR in Robert Debré hospital (01 June 2020) and (ii) mPCR use in Necker-Enfants malades hospital (01 June 2021). We included all children (0–17 years old) who visited these hospitals, with an mPCR test positive for at least one *E. coli* pathotype detected in the gastrointestinal syndromic panels used in the study, i.e., EAEC*,* EPEC*,* ETEC, STEC, and *Shigella/*EIEC, regardless of clinical presentation. Stool specimens were tested with the FilmArray gastrointestinal panel (BioMerieux, Salt Lake City, UT, USA) in Robert-Debré hospital or with the Bacterial GE+ assay (Novodiag, Copan, Italia) in Necker-Enfants malades hospital (Table 1). Cases were identified using the microbiological laboratory informatics systems from each center. This retrospective observational study was registered to our local Institutional Review Board (APHP) in July 2022 (register number 2022 0729163831).

**TABLE 1:** Pathogen detection by BioFire FilmArray gastrointestinal panel and Novodiag bacterial GE+

Pathogen	Novodiag bacterial GE+	BioFire FilmArray gastrointestinal panel
Bacteria		
*Campylobacter coli* or *jejuni*	+	+
Enteroaggregative *E. coli (EAEC*)	+	+
Enteropathogenic *E. coli (EPEC*)	+	+
*Enterotoxigenic E. coli (ETEC*)	+	+
Shiga toxin-producing *E. coli* (*STEC*)	+	+
*Shigella/enteroinvasive E. coli (EIEC*)	+	+
*Vibrio cholera or parahaemolyticus*	+	+
*Yersinia enterocolitica*	+	+
*Clostridium difficile* (toxin B gene)	+	+
*Salmonella*	+	+
*Yersinia pseudotuberculosis/pestis*	+	-
*Campylobacter upsaliensis*	-	+
*Clostridioides difficile* (toxin A)	-	+
*Plesiomonas shigelloides*	-	+
*E. coli* O157	-	+
*Vibrio vulnificus*	-	+
Parasites		
*Cryptosporidium spp*	-	+
*Cyclospora cayetanensis*	-	+
*Entamoeba histolytica*	-	+
*Giardia lamblia*	-	+
Viruses		
*Adenovirus* F40/41	-	+
*Astrovirus*	-	+
*Norovirus* GI/GII	-	+
*Rotavirus* A	-	+
*Sapovirus* (I, II, I,V and V)	-	+

### Data collection and definitions

Microbiological data regarding the identified *E. coli* and other potential pathogens (e.g., bacteria, viruses, parasites) detected by mPCR, as well as associated standard stool cultures used for bacterial confirmation, antigenic viral tests, or specific viral PCRs, were collected from the microbiological database of each participating center. In both centers, a standard stool culture was performed in cases where *Shigella*/EIEC, *Salmonella*, *Campylobacter,* or *Yersinia* were detected by mPCR. All clinical data were obtained from medical chart review. Patient demographic characteristics (age and sex), underlying chronic disease (i.e., immune deficiency, chronic gastrointestinal disease, recent abdominal surgery, or any disease requiring regular hospital follow-up), and recent travel (last 3 months) abroad were collected. Underlying chronic diseases comprised immunosuppression (primary immune deficiencies, active malignant conditions, evolutive neutropenia, uncontrolled HIV infection, or patients under immunosuppressive therapy), chronic gastrointestinal disease requiring specialized care (such as inflammatory bowel disease, exudative enteropathy, short bowel syndrome), recent abdominal surgery, or any patient with a disease requiring regular hospital follow-up. Clinical characteristics of the infectious episode, including fever [temperature of 38°C or higher (9)], diarrhea [defined as three or more loose or liquid stools per day for 3–14 days (10)], bloody diarrhea, persistent diarrhea [lasting more than 15 days (11)], and hospitalization, were recorded. Biological features, such as the highest leukocyte count and CRP value within 72 hours of stool collection, were documented. Additionally, antimicrobial therapy initiated for the current gastrointestinal infection and any modification made based on microbiological results were collected. Data on previous antimicrobial therapy initiated in the month prior to the mPCR were gathered through a review of medical charts; in cases where clinicians did not report any data on treatment, it was assumed that no antibiotics had been prescribed within the last month. In addition, we recorded the potential intervention of the local antimicrobial stewardship (AMS) team (12).

The appropriateness of antibiotic therapies following microbial identification was retrospectively assessed by E.B. and J.T. for the purpose of this study. These encompassed treatments initiated right after mPCR results and empiric treatments modified after mPCR results. An antibiotic treatment was deemed appropriate if it targeted ETEC in traveler's diarrhea infection or *Campylobacter* as outlined by both French and European guidelines (13, 14). Antibiotic treatment was also considered appropriate when initiated for a *Salmonella* spp. infection, either in infants <3 months old, immunocompromised patients, signs of sepsis, or recent travel to an area with high prevalence of *Salmonella* Typhi (13, 14). For this study, antibiotic treatment was considered appropriate for STEC detections only in young children under 3 years old or in immunocompromised patients, based on certain recommendations from national experts (Haut Conseil de Santé Publique 2015, available on https://www.hcsp.fr/explore.cgi/avisrapportsdomaine?clefr=495) (15). Antibiotic treatment for *Shigella*/EIEC was defined as appropriate in the following cases: severe cases requiring hospitalization, as antibiotic treatment may help reduce disease severity, shorten the length of stay, and lower the risk of secondary cases in the hospital, or positive *Shigella* cultures (regardless of severity or hospitalization) as this situation suggests an increased risk of person-to-person transmission due to a higher bacterial load in the feces. In case of EPEC, EAEC (14), or *Yersinia* detection (Center for Disease Control and Prevention guidelines on *Yersinia* treatment available on https://www.cdc.gov/yersinia/about/index.html), antibiotic therapy was always considered inappropriate, except in cases of co-detection with a pathogen requiring antibiotic initiation, as mentioned above.

### Statistical analyses

We first assessed the prevalence of *E. coli* detection in stool specimens of children tested with mPCR assays and described the potential co-detection of other pathogens. We compared general, clinical, and biological characteristics among cases based on the pathotype of *E. coli*. Next, we assessed the association between *E. coli* detection and treatment modifications, including antimicrobial initiation, switch, or discontinuation. In case of co-detection with several *E. coli* pathotypes in the same mPCR, each *E. coli* detection was evaluated separately. Categorical data were presented as numbers and proportions, whereas continuous variables were presented as mean [and standard deviation (SD)] or median [interquartile range (IQR) and range]. Clinical and biological characteristics of cases were compared among EAEC, EPEC, EIEC/*Shigella*, ETEC, and STEC cases using Fisher exact or Chi-squared tests for qualitative variables or analysis of variance (ANOVA) or Mann-Whitney U tests for quantitative variables. A difference was considered statistically significant if the *P* value was under 0.05. Statistical analyses were performed using Medistica software (available on https://www.pvalue.io/fr).

## RESULTS

### mPCR test results

**Fig 1:**
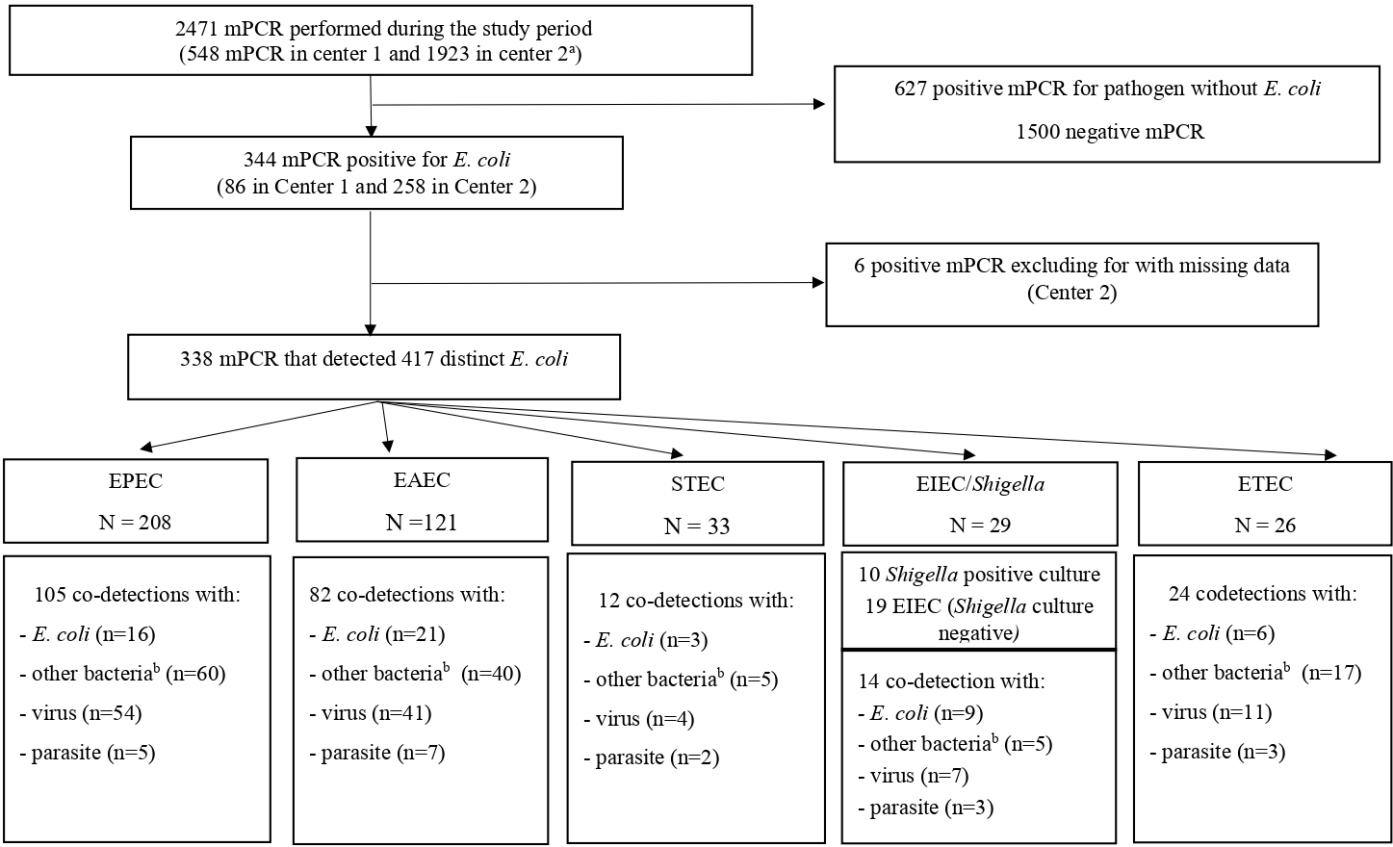
Flowchart of gastrointestinal mPCR results. ^a^mPCR in Center 1 is bacterial GE+ assay (Novodiag, Copan, Italia). mPCR in Center 2 is film array gastrointestinal panel (BioMerieux, Salt Lake City, UT, USA). ^b^Other bacteria detected in mPCR included *Campylobacter jejuni*, *Clostridioides difficile*, *Salmonella* spp., *Shigella*, *Plesiomonas shigelloides*, and *Yersinia enterocolitica*. EAEC: enteroaggregative *Escherichia coli*; STEC Shiga toxin-producing *E. coli*; EPEC enteropathogen *E. coli*; ETEC enterotoxinogen *E. coli*; EIEC enteroinvasive *E. coli*.

Between 01 June 2020 and 31 December 2021, 2,471 mPCRs were performed within the two participating hospitals. Among the 971 (39%) positive mPCRs, 344 (14%) tested positive for at least one *E. coli* pathotype with a similar proportion across the two centers [15.4% vs 13.7% (*P* = 0.18); Table S1] and 627 (25%) for another pathogen than *E. coli*. Within the 344 cases with *E. coli*, six were excluded due to missing data (Fig. 1). The remaining 338 mPCR tests corresponded to 338 distinct patients and detected 417 *E. coli* across the five pathotypes. The most prevalent pathotypes were EPEC (*n* = 208) and EAEC (*n* = 121). Standard stool culture was performed for the 29 mPCRs positive for EIEC/*Shigella*, and 10 of them (35%) isolated *Shigella* spp.

An *E. coli* was detected together with another microorganism in 161/338 (48%) cases, with a maximum of six co-detections in one mPCR. Viruses were co-detected in 105/338 cases (31%) and another bacterium in 91/338 cases (27%) (Table 2). The occurrence of a co-detection was associated with the pathotype of *E. coli* (*P* < 0.001) and was particularly frequent in ETEC (*n* = 24/26, 92%) and EAEC (*n* = 82/121, 68%) cases.

**TABLE 2:** mPCRs positive for *E. coli* and presentation of co-detected microbial agents^*e*^

N,%	Total of mPCRs (*n* = 338)	EPEC (*n* = 208)	EAEC (*n* = 121)	STEC (*n* = 33)	EIEC / Shigella (*n* = 29)	ETEC (*n* = 26)
Co-detection of ≥1 other microbial agent	161 (48)	105 (50)	82 (68)	12 (36)	24 (83)^*a*^	24 (92)
*E. coli* co-detections exclusively	54 (16)	16 (8)	21 (17)	3 (9)	8 (28)	6 (23)
EPEC	16 (5)	N/A	14 (12)	1 (3)	3 (10)	2 (8)
EAEC	21 (6)	13 (6)	N/A	0	6 (21)	4 (15)
STEC	3 (1)	0	2 (2)	N/A	1 (3)	1 (4)
EIEC	8 (2)	2 (1)	6 (5)	1 (3)	N/A	1 (4)
ETEC	6 (2)	3 (1)	4 (3)	1 (3)	1 (3)	N/A
Other bacteria	91 (27)	60 (26)	40 (33)	5 (15)	15 (55)	17 (65)
*Campylobacter jejuni*	29 (9)	23 (11)	11 (9)	1 (3)	2 (7)	5 (19)
*Clostridioides difficile*	16 (4)	9 (4)	3 (3)	3 (9)	0	1 (4)
*Salmonella* sp.	15 (4)	9 (4)	5 (4)	1 (3)	1 (3)	4 (15)
*Shigella*	10 (3)	3 (1)	4 (3)	0	10 (35)	2 (8)
*Plesiomonas shigelloides^b^*	5 (2)	4 (2)	4 (3)	0	2 (7)	1 (4)
*Yersinia enterocolitica*	1 (0)	1 (0)	1 (1)	0	0	0
Viruses	105 (31)	54 (26)	41 (34)	4 (12)	7 (24)	11 (42)
Rotavirus^*c*^	36 (11)	23 (11)	12 (10)	3 (9)	1 (3)	4 (15)
Norovirus^*d*^	25 (7)	14 (7)	17 (14)	0	4 (14)	3 (12)
Adenovirus^*c*^	19 (6)	11 (5)	10 (8)	1 (3)	1 (3)	4 (15)
Sapovirus^*b*^	14 (4)	7 (3)	9 (7)	0	0	0
Astrovirus^*b*^	11 (3)	8 (4)	5 (4)	0	1 (3)	2 (8)
Parasites^*b*^	14 (6)	5 (2)	7 (6)	2 (6)	3 (10)	3 (12)
*Giardia* spp.	8 (2)	2 (1)	4 (3)	1 (3)	2 (7)	2 (8)
*Cryptosporidium* spp.	6 (2)	3 (1)	4 (3)	1 (3)	1 (3)	1 (4)

^*a*^ Including the 10 *Shigella* detection in stool culture.

^*b*^ Available for 258 cases (mPCR results from FilmArray gastrointestinal panel).

^*c*^ Available for 317 cases (258 mPCR results from FilmArray gastrointestinal panel or from 59 antigenic tests).

^*d*^ Available for 275 cases (258 mPCR results from FilmArray gastrointestinal panel and 17 results from specific PCR).

^*e*^ N/A, not applicable; EAEC, enteroaggregative *Escherichia coli*; STEC, Shiga-toxin producing *E. coli*; EPEC, enteropathogen *E. coli* ; ETEC, enterotoxinogen *E. coli*; EIEC, enteroinvasive *E. coli*; mPCR, syndromic multiplex PCR.

### Study population and general characteristics

Among the 338 positive mPCRs, 158 (47%) were from patients with an underlying chronic disease (Table 3). The mean age of patients was 4.2 [SD 4.8, range (0–17)] years, and the majority of them were males (61%). All 128/338 (38%) non-hospitalized cases reported moderate diarrhea symptoms and were subsequently managed at home following the emergency department visit. Of the 210/338 (62%) patients who were hospitalized, 138/210 (66%) were initially admitted due to gastrointestinal symptoms. The primary indication for mPCR testing was diarrhea (*n* = 269/338, 80%), including cases of bloody diarrhea (*n* = 65/338, 19%), traveler's diarrhea (*n* = 68/338, 20%), and persistent diarrhea (*n* = 5/338, 2%). For the 69 (20%) remaining cases where the indication was not diarrhea, mPCR was prescribed in patients with abdominal pain [returning travel (*n* = 4) or not (*n* = 18)], bloody stool without diarrhea (*n* = 6), fever of unknown origin (*n* = 5), other non-diarrheal symptoms [neurological (*n* = 2), respiratory (*n* = 6), other symptoms (*n* = 14)] or in asymptomatic patients (*n* = 14). Among asymptomatic patients, testing was conducted during migrant or post-travel consultation (*n* = 4), in allergological assessment or pretherapeutic screening (*n* = 6), and in follow-up screening related to previous digestive (*n* = 2) or neurologic symptoms (*n* = 2). Fever was observed in 141/338 (42%) patients.

**TABLE 3:** Clinical data and characteristics of *E. coli* pathotypes^*g*^

N, %	All mPCRs (*n* = 338)^*a*^	EPEC (*n* = 208)	EAEC (*n* = 121)	STEC (*n* = 33)	EIEC/*Shigella* (*n* = 29)	ETEC (*n* = 26)	*P* ^*f*^
Centers							
Center 1	86 (25)	57 (27)	29 (24)	5 (15)	7 (24)	4 (15)	
Center 2	252 (75)	151 (73)	92 (76)	28(85)	22 (76)	22 (85)	
General characteristics of patients							
Age, year; mean (SD), [range]	4.0 (4.8), [0–17]	4.0 (4.9), [0–17]	3.8 (4.4), [0–17]	5.2 (5.6), [0–15]	5.5 (3.6), [0–13]	5.0 (4.9), [0–16]	**0.02**
Sex ratio (M/F)	206/132	119/89	77/44	20/13	18/11	20/6	0.41
Underlying chronic disease	158 (47)	106 (51)	53 (44)	11 (33)	4 (14)	10 (38)	**0.002**
Gastrointestinal disease^*b*^	60 (18)	42 (20)	16 (13)	4 (12)	0	3 (12)	
Immunosuppression^*c*^	49 (14)	43 (20)	22 (18)	5 (15)	1 (3)	1 (4)	
Other^*d*^	49 (14)	21 (10)	15 (12)	2 (6)	3 (10)	6 (23)	
Previous antimicrobial therapy	29 (9)	17 (9)	11 (9)	3 (8)	3 (10)	0	0.06
Recent travel (< month)	68 (20)	33 (16)	44 (36)	4 (12)	21 (72)	14 (54)	**<0.001**
Africa	61	30	42	2	20	13	
Asia	4	2	0	1	1	1	
Other	3	1	2	1	0	0	
Clinical characteristics at the time of mPCR testing							
Diarrhea	269 (80)	168 (81)	99 (82)	23 (70)	25 (86)	21 (81)	0.54
Bloody diarrhea	65 (19)	40 (19)	21 (17)	5 (15)	8 (28)	2 (8)	
Fever	141 (42)	86 (41)	49 (41)	17 (52)	12 (41)	9 (35)	0.86
Abdominal pain	134 (40)	70 (34)	50 (41)	21 (64)	17 (59)	13 (50)	**0.001**
No symptoms^*e*^	14 (5)	9 (5)	3 (2)	2 (6)	2 (7)	1 (4)	0.6
Biological data							
CRP, mg/L; mean (SD)	41.1 (0–103.7)	34.5 (0–86.7)	33.5 (0–82.1)	75.8 (0–185.8)	76.8 (0–170.4)	33.8 (0–75.8)	0.49
Leukocytes (G/L); mean (SD)	11.0 (4.7–16.9)	11.1 (4.5–17.7)	10.2 (4.4–16.0)	11.0 (5.0–16.0)	11.3 (6.4–16.2)	10.0 (4.8–15.2)	0.77
Antibiotic therapy							
Empirical treatment before mPCR results	67 (20)	40 (19)	21 (17)	7 (21)	8 (28)	5 (19)	0.8
Changes following mPCR results	10 (15)	4 (2)	1 (1)	4 (12)	1 (4))	0	
Additional antibiotic	7 (2)	2 (1)	1 (1)	4 (12)	0	N/A	
Antibiotic switch	3 (1)	2 (1)	0	0	1 (4	N/A	
Discontinuation	0	0	0	0	0	N/A	
Initiation following mPCR results	69 (20)	35 (17)	24 (20)	16 (48)	14 (48)	8 (31)	**<0.001**
Appropriate	38 (11)	19 (9)	15 (12)	8 (24)	8 (27)	7 (27)	
Co-detection with *Campylobacter,* *Salmonella, or Shigella* spp.	25 (7)	17 (8)	10 (8)	1 (3)	3 (10)	4 (15)	
Inappropriate	31 (9)	16 (8)	9 (7)	8 (24)	6 (21)	1 (4)	0.4
Co-detection with virus, *Pleisiomonas shigelloides,* *Cryptosporidium,* non-Typhi *Salmonella*	10 (3)	3 (1)	4 (3)	4 (12)	1 (3)	1 (4)	

^*a*^ In case of co-detection of several *E. coli* pathotypes in a same PCR, each pathogen detected was analyzed separately.

^*b*^ Crohn’s disease (*n* = 10) or other inflammatory bowel disease (*n* = 20), Hirschsprung syndrome (*n* = 16), malabsorption enteropathy (*n* = 6), gastrointestinal malformation (*n* = 2), others (*n* = 6).

^*c*^ Systemic disease with immunosuppressive treatment (*n* = 26), malignant blood diseases or cancer (= 10), solid organ transplant recipient (*n* = 13);

^*d*^ Sickle cell disease (*n* = 17), extreme prematurity (*n* = 7), genetic disorder (*n* = 7), congenital anomalies of the kidney and urinary tract (*n* = 9), other (*n* = 9);

^*e*^ Asymptomatic migrant or post-travel consultations (*n* = 4), allergological assessment or pretherapeutic screening (*n* = 6), follow-up screening related to previous digestive (*n* = 2) or neurologic symptoms (*n* = 2).

^*f*^ Variables were compared between *Escherichia coli* pathotypes, and *P* value <0.05 was considered significant.

^*g*^ EAEC, enteroaggregative *E. coli*; STEC Shiga toxin-producing *E. coli*; EPEC enteropathogen *E. coli*; ETEC enterotoxinogen *E. coli*; EIEC enteroinvasive *E. coli*.

Except for abdominal pain, which was associated with the *E. coli* pathotype (*P* = 0.001), clinical features were similar across *E. coli* pathotypes. Recent travel was more often reported in patients without any chronic underlying disease (84% vs. 46%, *P* < 0.001) and who had more than one pathogen identified (75%, *P* < 0.001). Moreover, travel was associated with the *E. coli* pathotype (*P* < 0.001) and was particularly frequent with EIEC/*Shigella* detection (72%). Three patients died within the 15 days following *E. coli* detection. The deaths were not related to *E. coli* infection but resulted from an invasive pneumococcal infection, cardiac arrest caused by arrhythmia, and Reye's syndrome.

### Antimicrobial treatment and appropriateness

A total of 136/338 (40%) cases were treated with antibiotics, including 67 treatments initiated empirically before and 69 initiated following mPCR results (Table 3). Regarding the 67 empirical treatments, no antibiotic treatment was discontinued, and 10 (15%) were modified following the positive mPCR result: azithromycin was added in seven cases (four cases with STEC, one with EPEC, and two bacterial co-detection with *C. jejuni* and *Salmonella*), and one treatment was switched from amoxicillin to cefpodoxime following an EPEC detection. Two patients with recent travel had their initial therapy switched from an intravenous (IV) third-generation cephalosporin to azithromycin following EPEC and EIEC/*Shigella* detection, respectively.

Among the 69 treatments initiated after mPCR results, azithromycin was the most common (*n* = 57, 83%) antimicrobial agent, followed by intravenous third-generation cephalosporins (*n* = 6, 9%), vancomycin (*n* = 2, 3%), metronidazole (*n* = 9, 13%), and cotrimoxazole (*n* = 1). These treatments were mainly initiated in the context of co-detection with *Campylobacter jejuni* (*n* = 29, 9%), *Clostridioides difficile* (*n* = 16, 4%), *Salmonella* (*n* = 15, 4%), and *Shigella* (*n* = 10, 3%). In 34 cases (49%), the treatment was introduced for *E. coli* alone (Table 3). EPEC and EAEC cases were detailed in Table S2. The AMS team intervened in 40/338 (12%) cases at the clinicians' request, with 32 of these being inpatients, resulting in the following recommendations: initiation of antibiotics in 15 cases, no indication for treatment in 14 cases, switching or discontinuation of antibiotics in four cases, and continuation of current treatment in seven cases.

When each case was retrospectively reviewed and reassessed, 31/69 (45%) of antibiotic therapies initiated after the mPCR results were considered inappropriate according to the definitions described above (Table 3). In 21 cases, the treatment was initiated solely for *E. coli* detections (13 EPEC, five EAEC, four STEC, or five EIEC, including *E. coli* co-detections). The last 10 cases were of *E. coli* associated with co-pathogens not requiring antibiotics (Table 3). Upon retrospective review of the 40 AMS audits, nine cases of azithromycin initiation were found to be inappropriate according to guidelines. Among the 158 patients with underlying conditions, 72 (45%) received antibiotic treatment, with 47 of them (65%) deemed overtreated.

## DISCUSSION

### Main findings

This is the first study aiming to describe cases of infections with *E. coli* pathotypes identified using gastrointestinal mPCRs in children. As *E. coli* pathotypes are not typically detected in standard stool culture, and their role in disease is debated, these identifications may influence antimicrobial prescriptions. We observed that *E. coli* was frequently identified by mPCRs (14% of the 2,471 mPCR performed), with a high rate of co-detection (48%) with digestive viruses or bacterial pathogens. Many antibiotic treatments initiated after the mPCR results (*n* = 31/69, 45%) were deemed inappropriate.

### Interpretation

Previous studies have shown that the mPCR (Filmarray BioFire, BioMerieux) could detect at least one pathogen in 70–98% of children with bloody or traveler's diarrhea (3, 4) with an increased detection rate by 25–50% with mPCRs compared to conventional methods (3, 16). Although the *E. coli* pathotypes could contribute to the development of diarrhea (17, 18), their clinical expression and severity vary widely among studies (4, 19–21). In our study, we also identified positive mPCRs for *E. coli* in few asymptomatic patients (*n* = 14, 4%), though most mPCRs were performed in symptomatic patients. Previous works also showed *E. coli* detections in asymptomatic children; two recent studies detected 0.5–1% of STEC in stool specimens of asymptomatic children (22, 23) and a prevalence of EPEC carriage reaching 10–20% (19, 20, 24). This clinical variability might be explained by host susceptibility (25), along with the impact of virulence factors of *E. coli* undetected by mPCRs (17, 26), such as *bfp* for EPEC (17). Moreover, we cannot exclude the possibility that some detections are false positive results (e.g., PCR target transmitted by plasmid to other bacteria), especially in asymptomatic children. Previous studies have showed that the mPCR tests demonstrate good specificity compared to standard molecular methods (27, 28). The mPCRs used in the study could not provide cycle threshold values (representative of bacterial load), which may also correlate with clinical presentation and disease severity (19). Co-detection of other pathogens might also explain the symptoms. This co-detection was frequent in our study (48%) involving pathogenic viruses such as rotavirus, norovirus, or sapovirus or bacteria such as *Campylobacter* and *Salmonella* spp. However, prolonged excretion has been described for *Salmonella* (29) or STEC (30), and they might be detected even if unrelated to symptoms. Overall, interpreting *E. coli* detection is challenging and requires consideration of all identified pathogens, the patient's underlying diseases and travels, and the consequences of unnecessary antibiotic therapy when deciding on treatment.

We observed a substantial rate of inappropriate therapy following the detection of *E. coli* (for 45% of the 69 introduced antibiotics). Interestingly, one study in adults showed that the use of gastrointestinal mPCR leads to a decrease in antimicrobial prescriptions (14.5% of discontinuation versus 4.5% in the standard stool culture group, *P* < 0.01) (31). Conversely, a randomized controlled trial on adults showed that routine molecular point-of-care testing for gastrointestinal pathogens, despite improving time to results and reducing time spent in single-occupancy isolation rooms, was also accompanied by increased antibiotic use (32). In addition, a previous French pediatric study showed that antibiotic therapy was initiated in 16.3% of cases after a positive mPCR result, and only 0.6% of cases saw antibiotics discontinued (4). Here, many cases with EPEC and EAEC detection received antibiotic treatment, suggesting that a positive result, even for weakly pathogenic bacteria (33), might lead to the prescription of antibiotics despite available guidelines (13, 15). The AMS team conducted few prescription reviews (12%), and several were retrospectively deemed inappropriate despite existing recommendations. This underscores the potential inconsistencies in the literature and the challenges in applying guidelines based on microbiological results, even by experts like the AMS team, especially in facilities caring for patients with significant comorbidities. The limited involvement of the AMS team here was primarily due to reviews being conducted only at the clinicians' request. To address these issues, alternative strategies such as algorithms and decision-support tools developed and validated by the AMS team in collaboration with microbiologists and clinicians could improve management. Furthermore, a patient's vulnerability associated with an underlying condition (such as immunodeficiency or chronic gastrointestinal disease) or a severe clinical presentation may influence the clinician's decision to initiate antibiotic treatment rather than searching for an alternative diagnosis, as we observed 65% of over-treatment in this population in our study. As mPCRs do not provide information on antibiotic susceptibility, empiric broad-spectrum antibiotics might be employed. In this study, azithromycin was the most used antibiotic for bacterial diarrhea. Even if this antibiotic is usually prescribed for a short course (3 days), its long-term impact on intestinal microbiota composition and richness needs thoughtful evaluation before prescription (34, 35).

The detection of *E. coli* pathotypes could be particularly valuable in food-borne illness, persistent diarrhea, or immunocompromised children without any other diagnosis (36). In addition, mPCR could improve the diagnosis of inflammatory bowel disease in children by broadly ruling out infection when facing prolonged gastrointestinal symptoms; indeed, usually, at the time of inflammatory bowel disease diagnosis, a third of patients present with febrile diarrhea, which may erroneously suggest an infectious cause (37). To prevent inappropriate treatment, one suggestion could be that microbiologists partially disclose mPCR results using flexible testing and reporting options based on the patient's background or clinical presentation. For instance, detection of EPEC and EAEC could be revealed upon request only in cases of diarrhea occurring in high-risk populations (e.g., immunocompromised individuals, very young infants) or patients with severe symptoms without any other identifiable cause. Furthermore, the development of mPCR testing strategies targeting only the most virulent pathogens may also be beneficial.

### Limitations

Our study has several limitations. First, data were collected from a retrospective review of medical records. Although antibiotic treatment was mainly reported in medical charts, we could not rule out that some treatments were not recorded. Second, the indication of treatment was not always detailed, which may have affected our retrospective assessment of the appropriateness of antibiotic therapy. In addition, we did not perform multivariate analyses on the different variables studied since our main objective was to describe the population and not to search for factors associated with the mPCR results. Finally, this study did not evaluate the effectiveness of antibiotic treatment for *E. coli* infection on symptoms and outcomes of patients nor did it assess the overall impact of mPCR on patient care, due to the descriptive design. A randomized controlled trial with antibiotic use as the primary endpoint is needed to complement previous trials that mainly focused on time to result availability and did not specifically address the issue of *E. coli* pathotypes (38, 39). However, our study is, to our knowledge, the largest cohort studying the use of mPCR, the detection of *E. coli,* and therapeutic consequences.

### Conclusion

Digestive mPCR is a recent diagnosis technique that has gained increased use in hospital laboratories due to its high detection rate and rapid results. The large number of inappropriate antibiotic treatments found in this study raises questions about the necessity of restricting these tests using clinical decision support systems or disclosing their results only in specific situations, with the collaboration of infectious diseases specialists and microbiologists. Further prospective studies may be of interest to assess the benefits and risks of these mPCRs in patient care.

## Data Availability

All clinical and microbiological data supporting the results of this study are included within the manuscript. The anonymized database, containing individual data for each case, will be made accessible upon reasonable request.

## SUPPLEMENTAL MATERIAL

Supplemental tables jcm.01073-24-s0001.docx Tables S1 and S2.

ASM does not own the copyrights to Supplemental Material that may be linked to, or accessed through, an article. The authors have granted ASM a non-exclusive, world-wide license to publish the Supplemental Material files. Please contact the corresponding author directly for reuse.

## References

[1] Florez ID, Niño-Serna LF, Beltrán-Arroyave CP. 2020. Acute infectious diarrhea and gastroenteritis in children. Curr Infect Dis Rep 22:4. doi:10.1007/s11908-020-0713-6 31993758

[2] Stockmann C, Pavia AT, Graham B, Vaughn M, Crisp R, Poritz MA, Thatcher S, Korgenski EK, Barney T, Daly J, Rogatcheva M. 2017. Detection of 23 gastrointestinal pathogens among children who present with diarrhea. J Pediatric Infect Dis Soc 6:231–238. doi:10.1093/jpids/piw020 27147712 PMC5907859

[3] Pouletty M, De Pontual L, Lopez M, Morin L, Poilane I, Pham LL, Carbonnelle E, Titomanlio L, Faye A, Bonacorsi S. 2019. Multiplex PCR reveals a high prevalence of multiple pathogens in traveller’s diarrhoea in children. Arch Dis Child 104:141–146. doi:10.1136/archdischild-2017-314327 29982173

[4] Truong J, Cointe A, Le Roux E, Bidet P, Michel M, Boize J, Mariani-Kurkdjian P, Caseris M, Hobson CA, Desmarest M, Titomanlio L, Faye A, Bonacorsi S. 2022. Clinical impact of a gastrointestinal PCR panel in children with infectious diarrhoea. Arch Dis Child 107:601–605. doi:10.1136/archdischild-2021-322465 34921002

[5] Zhang H, Morrison S, Tang Y-W. 2015. Multiplex polymerase chain reaction tests for detection of pathogens associated with gastroenteritis. Clin Lab Med 35:461–486. doi:10.1016/j.cll.2015.02.006 26004652 PMC5002946

[6] Beal SG, Tremblay EE, Toffel S, Velez L, Rand KH. 2018. A gastrointestinal PCR panel improves clinical management and lowers health care costs. J Clin Microbiol 56:e01457-17. doi:10.1128/JCM.01457-17 29093106 PMC5744222

[7] Axelrad JE, Freedberg DE, Whittier S, Greendyke W, Lebwohl B, Green DA. 2019. Impact of gastrointestinal panel implementation on health care utilization and outcomes. J Clin Microbiol 57:e01775-18. doi:10.1128/JCM.01775-18 30651393 PMC6425162

[8] Özcan N, Bacalan F, Çakır F, Bilden A, Genişel N, Dal T. 2022. Culture and culture-independent diagnostic tests in Campylobacter enteritis. J Infect Dev Ctries 16:616–621. doi:10.3855/jidc.14902 35544622

[9] Mackowiak PA, Chervenak FA, Grünebaum A. 2021. Defining fever. Open Forum Infect Dis 8:ofab161. doi:10.1093/ofid/ofab161 34476283 PMC8394829

[10] World Health Organization. 2005. Definition of diarrhoea, p 4. In WHO, the treatment of diarrhoea: a manual for physicians and other senior health workers

[11] Bandsma RHJ, Sadiq K, Bhutta ZA. 2019. Persistent diarrhoea: current knowledge and novel concepts. Paediatr Int Child Health 39:41–47. doi:10.1080/20469047.2018.1504412 30079818

[12] Poline J, Postaire M, Parize P, Pilmis B, Bille E, Zahar JR, Frange P, Cohen JF, Lortholary O, Toubiana J. 2021. Stewardship program on carbapenem prescriptions in a tertiary hospital for adults and children in France: a cohort study. Eur J Clin Microbiol Infect Dis 40:1039–1048. doi:10.1007/s10096-020-04103-3 33389261 PMC7778866

[13] Cohen R, Raymond J, Gendrel D. 2017. Antimicrobial treatment of diarrhea/acute gastroenteritis in children. Arch Pediatr 24:S26–S29. doi:10.1016/S0929-693X(17)30515-8 29290231

[14] Guarino A, Ashkenazi S, Gendrel D, Lo Vecchio A, Shamir R, Szajewska H, European Society for Pediatric Gastroenterology, Hepatology, and Nutrition, European Society for Pediatric Infectious Diseases. 2014. European Society for Pediatric Gastroenterology, Hepatology, and Nutrition/European Society for Pediatric Infectious Diseases evidence-based guidelines for the management of acute gastroenteritis in children in Europe: update 2014. J Pediatr Gastroenterol Nutr 59:132–152. doi:10.1097/MPG.0000000000000375 24739189

[15] Kakoullis L, Papachristodoulou E, Chra P, Panos G. 2019. Shiga toxin-induced haemolytic uraemic syndrome and the role of antibiotics: a global overview. J Infect 79:75–94. doi:10.1016/j.jinf.2019.05.018 31150744

[16] Calderaro A, Martinelli M, Buttrini M, Montecchini S, Covan S, Rossi S, Ferraglia F, Montagna P, Pinardi F, Larini S, Arcangeletti MC, Medici MC, Chezzi C, De Conto F. 2018. Contribution of the FilmArray^®^ Gastrointestinal Panel in the laboratory diagnosis of gastroenteritis in a cohort of children: a two-year prospective study. Int J Med Microbiol 308:514–521. doi:10.1016/j.ijmm.2018.04.007 29748124

[17] Denamur E, Clermont O, Bonacorsi S, Gordon D. 2021. The population genetics of pathogenic Escherichia coli. Nat Rev Microbiol 19:37–54. doi:10.1038/s41579-020-0416-x 32826992

[18] Kotloff KL, Nataro JP, Blackwelder WC, Nasrin D, Farag TH, Panchalingam S, Wu Y, Sow SO, Sur D, Breiman RF, et al.. 2013. Burden and aetiology of diarrhoeal disease in infants and young children in developing countries (the Global Enteric Multicenter Study, GEMS): a prospective, case-control study. Lancet 382:209–222. doi:10.1016/S0140-6736(13)60844-2 23680352

[19] Levine MM, Robins-Browne RM. 2012. Factors that explain excretion of enteric pathogens by persons without diarrhea. Clin Infect Dis 55 Suppl 4:S303–S311. doi:10.1093/cid/cis789 23169942 PMC3502317

[20] Alikhani MY, Mirsalehian A, Fatollahzadeh B, Pourshafie MR, Aslani MM. 2007. Prevalence of enteropathogenic and shiga toxin-producing Escherichia coli among children with and without diarrhoea in Iran. J Health Popul Nutr 25:88–93. 17615908 PMC3013268

[21] Khairy RMM, Fathy ZA, Mahrous DM, Mohamed ES, Abdelrahim SS. 2020. Prevalence, phylogeny, and antimicrobial resistance of Escherichia coli pathotypes isolated from children less than 5 years old with community acquired- diarrhea in Upper Egypt. BMC Infect Dis 20:908. doi:10.1186/s12879-020-05664-6 33256619 PMC7708180

[22] Bizot E, Cointe A, Béchet S, Sobral E, Cohen R, Mariani-Kurkdjian P, Levy C, Bonacorsi S. 2021. Shiga toxin-producing Escherichia coli carriage in 959 healthy French infants. Arch Dis Child 106:1239–1240. doi:10.1136/archdischild-2021-321601 33832927

[23] Harries M, Dreesman J, Rettenbacher-Riefler S, Mertens E. 2016. Faecal carriage of extended-spectrum β-lactamase-producing Enterobacteriaceae and Shiga toxin-producing Escherichia coli in asymptomatic nursery children in Lower Saxony (Germany), 2014. Epidemiol Infect 144:3540–3548. doi:10.1017/S0950268816001837 27608837 PMC9150205

[24] Enserink R, Scholts R, Bruijning-Verhagen P, Duizer E, Vennema H, de Boer R, Kortbeek T, Roelfsema J, Smit H, Kooistra-Smid M, van Pelt W. 2014. High detection rates of enteropathogens in asymptomatic children attending day care. PLoS ONE 9:e89496. doi:10.1371/journal.pone.0089496 24586825 PMC3933542

[25] Gallardo P, Izquierdo M, Vidal RM, Soto F, Ossa JC, Farfan MJ. 2020. Gut microbiota-metabolome changes in children with diarrhea by diarrheagenic E. coli. Front Cell Infect Microbiol 10:485. doi:10.3389/fcimb.2020.00485 33072619 PMC7531578

[26] Hazen TH, Donnenberg MS, Panchalingam S, Antonio M, Hossain A, Mandomando I, Ochieng JB, Ramamurthy T, Tamboura B, Qureshi S, Quadri F, Zaidi A, Kotloff KL, Levine MM, Barry EM, Kaper JB, Rasko DA, Nataro JP. 2016. Genomic diversity of EPEC associated with clinical presentations of differing severity. Nat Microbiol 1:15014. doi:10.1038/nmicrobiol.2015.14 27571975 PMC5067155

[27] Buss SN, Leber A, Chapin K, Fey PD, Bankowski MJ, Jones MK, Rogatcheva M, Kanack KJ, Bourzac KM. 2015. Multicenter evaluation of the BioFire FilmArray gastrointestinal panel for etiologic diagnosis of infectious gastroenteritis. J Clin Microbiol 53:915–925. doi:10.1128/JCM.02674-14 25588652 PMC4390666

[28] Jo SJ, Kang HM, Kim JO, Cho H, Heo W, Yoo IY, Park Y-J. 2021. Evaluation of the BioFire gastrointestinal panel to detect diarrheal pathogens in pediatric patients. Diagnostics (Basel) 12:34. doi:10.3390/diagnostics12010034 35054200 PMC8774520

[29] Buchwald DS, Blaser MJ. 1984. A review of human salmonellosis: II. Duration of excretion following infection with nontyphi Salmonella. Rev Infect Dis 6:345–356. doi:10.1093/clinids/6.3.345 6377442

[30] Nitschke M, Sayk F, Härtel C, Roseland RT, Hauswaldt S, Steinhoff J, Fellermann K, Derad I, Wellhöner P, Büning J, Tiemer B, Katalinic A, Rupp J, Lehnert H, Solbach W, Knobloch J-M. 2012. Association between azithromycin therapy and duration of bacterial shedding among patients with Shiga toxin-producing enteroaggregative Escherichia coli O104:H4. JAMA 307:1046–1052. doi:10.1001/jama.2012.264 22416100

[31] Torres-Miranda D, Akselrod H, Karsner R, Secco A, Silva-Cantillo D, Siegel MO, Roberts AD, Simon GL. 2020. Use of BioFire FilmArray gastrointestinal PCR panel associated with reductions in antibiotic use, time to optimal antibiotics, and length of stay. BMC Gastroenterol 20:246. doi:10.1186/s12876-020-01394-w 32727381 PMC7392718

[32] Brendish NJ, Beard KR, Malachira AK, Tanner AR, Sanga-Nyirongo L, Gwiggner M, Cummings JRF, Moyses HE, Clark TW. 2023. Clinical impact of syndromic molecular point-of-care testing for gastrointestinal pathogens in adults hospitalised with suspected gastroenteritis (GastroPOC): a pragmatic, open-label, randomised controlled trial. Lancet Infect Dis 23:945–955. doi:10.1016/S1473-3099(23)00066-X 37116527

[33] Hu J, Torres AG. 2015. Enteropathogenic Escherichia coli: foe or innocent bystander? Clin Microbiol Infect 21:729–734. doi:10.1016/j.cmi.2015.01.015 25726041 PMC4497942

[34] Wei S, Mortensen MS, Stokholm J, Brejnrod AD, Thorsen J, Rasmussen MA, Trivedi U, Bisgaard H, Sørensen SJ. 2018. Short- and long-term impacts of azithromycin treatment on the gut microbiota in children: a double-blind, randomized, placebo-controlled trial. EBioMedicine 38:265–272. doi:10.1016/j.ebiom.2018.11.035 30478001 PMC6306380

[35] McDonnell L, Gilkes A, Ashworth M, Rowland V, Harries TH, Armstrong D, White P. 2021. Association between antibiotics and gut microbiome dysbiosis in children: systematic review and meta-analysis. Gut Microbes 13:1–18. doi:10.1080/19490976.2020.1870402 PMC7928022 33651651

[36] Hu P, Liu C, Ruan J, Yuan M, Ju C, Ma Y, Yuan Y, Chen H, Yu M, Duan Y. 2020. FilmArray GI-panel performance for the rapid and multiple detection of gastrointestinal microorganisms in foodborne illness outbreaks in Shenzhen during 2018–2019. Infect Genet Evol 86:104607. doi:10.1016/j.meegid.2020.104607 33132110

[37] Rosen MJ, Dhawan A, Saeed SA. 2015. Inflammatory bowel disease in children and adolescents. JAMA Pediatr 169:1053–1060. doi:10.1001/jamapediatrics.2015.1982 26414706 PMC4702263

[38] DiDiodato G, Allen A, Bradbury N, Brown J, Cruise K, Jedrzejko C, MacDonald V, Pigeon J, Sturgeon A, Yellenik D. 2022. The efficacy of the BioFire filmarray gastrointestinal panel to reduce hospital costs associated with contact isolation: a pragmatic randomized controlled trial. Cureus 14:e27931. doi:10.7759/cureus.27931 36120274 PMC9464456

[39] Xie J, Kim K, Berenger BM, Chui L, Vanderkooi OG, Grisaru S, Freedman SB. 2023. Comparison of a rapid multiplex gastrointestinal panel with standard laboratory testing in the management of children with hematochezia in a pediatric emergency department: randomized controlled trial. Microbiol Spectr 11:e0026823. doi:10.1128/spectrum.00268-23 37039648 PMC10269456

