# Supplementary material for: Use of gastrointestinal syndromic multiplex molecular assays and detection of Escherichia coli pathotypes in pediatric wards
Source: J Clin Microbiol. 2025 Feb 26;63(4):e01073-24. doi: 10.1128/jcm.01073-24 (PMC11980392; doi:10.1128/jcm.01073-24)
Supplement: Supplemental tables — Tables S1 and S2. [file jcm.01073-24-s0001.docx]

**Supplementary Table 1. Distribution of *E. coli* pathotypes detected with gastrointestinal mPCRs performed**

| **N, %** | **Novodiag Bacterial GE+**  **N=548** | | **BioFire Filmarray panel**  **N=1923** | **Total**  **N=2471** |
| --- | --- | --- | --- | --- |
| **Number of mPCRs positive for *E. coli***  **(with or without copathogen)** | 86 (15.7) | 258 (13.4) | | 344 (13.9)^b^ |
| **Number of *E. coli* detected by the panels** | 102 | 321 | | 423 |
| - EAEC | 29 (5.3) | 92 (4.8) | | 121 (4.9) |
| - STEC | 5 (0.9) | 30 (1.6) | | 35 (1.4)^b^ |
| - EPEC | 57 (10.4) | 154 (8.0) | | 211 (8.5)^b^ |
| - ETEC | 4 (0.7) | 23 (1.2) | | 27 (1.1) ^b^ |
| - EIEC/*Shigella* | 7 (1.3) | 22 (1.1) | | 29 (1.2) |
| **Detection of another pathogen in mPCR (non *E. coli)*** | 72 (13.1) | 555 (28.9) | | 627 (25.4) |
| **Negative mPCR^a^** | 390 (71.2) | 1110 (57.7) | | 1500 (60.7) |

^a^Negative for *E. coli* or other bacterial, viral or parasitic tested pathogen; ^b^Among these cases, 6/344 mPCR results representing 3 EPEC, 2 STEC and 1 ETEC were excluded for further analyses, due to missing data. EAEC, enteroaggregative *E. coli*; STEC, Shiga-toxin producing *E. coli*; EPEC, enteropathogen *E. coli*; ETEC, enterotoxinogen *E. coli*; EIEC, Enteroinvasive *E. coli*

**Supplementary Table 2. Details of EPEC and EAEC cases (n=49) treated with antibiotics following mPCR results.**

| **Case** | **EPEC** | **EAEC** | **Other pathogen detected** | **Treatment** | **Underlying disease** | **Appropriateness^a^** |
| --- | --- | --- | --- | --- | --- | --- |
| ***1*** | **X** | **-** | - | Metronidazole | Yes | No |
| ***2*** | **X** | **-** | - | Azithromycin | Yes | No |
| ***3*** | **X** | **-** | - | Azithromycin | Yes | No |
| ***4*** | **X** | **-** | - | Azithromycin | Yes | No |
| ***5*** | **X** | **-** | - | Azithromycin | Yes | No |
| ***6*** | **X** | **-** | - | Cotrimoxazole | Yes | No |
| ***7*** | **X** | **-** | - | Azithromycin | Yes | No |
| ***8*** | **X** | **-** | - | Azithromycin | Yes | No |
| ***9*** | **X** | **-** | - | Azithromycin | Yes | No |
| ***10*** | **X** | **-** | - | Azithromycin + Amoxicillin - clavulanate | Yes | No |
| ***11*** | **X** | **-** | - | Azithromycin | Yes | No |
| ***12*** | **X** | **-** | - | Azithromycin | No | No |
| ***13*** | **-** | **X** | - | Azithromycin | No | No |
| ***14*** | **-** | **X** | - | Azithromycin | No | No |
| ***15*** | **X** | **X** | *C. jejuni* | Azithromycin | Yes | Yes |
| ***16*** | **X** | **X** | *C. jejuni* | Azithromycin | Yes | Yes |
| ***17*** | **X** | **X** | EIEC | Azithromycin | No | No |
| ***18*** | **X** | **X** | ETEC | Azithromycin | No | Yes |
| ***19*** | **X** | **X** | *Plesiomonas shigelloides* | Azithromycin | Yes | No |
| ***20*** | **X** | **X** | ETEC, *Cryptosporidium spp*, *Plesiomonas shigelloides* | Azithromycin | No | Yes |
| ***21*** | **X** | **X** | *Salmonella, Cryptosporidium spp , Astrovirus*, EIEC | Azithromycin | No | Yes |
| ***22*** | **X** | **X** | *C. jejuni* | Azithromycin | No | Yes |
| ***23*** | **X** | **X** | ETEC, *C. jejuni, Astrovirus* | Azithromycin | Yes | Yes |
| ***24*** | **X** | **X** | *Shigella* | Azithromycin | Yes | Yes |
| ***25*** | **X** | **-** | *C. difficile* | Vancomycin | Yes | Yes |
| ***26*** | **X** | **-** | *Rotavirus* | Azithromycin | Yes | No |
| ***27*** | **X** | **-** | *C. jejuni* | Azithromycin | No | Yes |
| ***28*** | **X** | **-** | *C. jejuni* | Azithromycin | No | Yes |
| ***29*** | **X** | **-** | *Giardia lamblia* | Metronidazole | Yes | Yes |
| ***30*** | **X** | **-** | *C. jejuni* | Azithromycin | No | Yes |
| ***31*** | **X** | **-** | *C. jejuni* | Azithromycin | Yes | Yes |
| ***32*** | **X** | **-** | *C. jejuni* | Azithromycin | No | Yes |
| ***33*** | **X** | **-** | *C. jejuni* | Azithromycin | No | Yes |
| ***34*** | **X** | **-** | *Adenovirus, C. jejuni, Giardia lamblia*, ETEC | Azithromycin + Metronidazole | No | Yes |
| ***35*** | **X** | **-** | *C. difficile* | Vancomycin | Yes | Yes |
| ***36*** | **X** | **-** | *C. jejuni, Norovirus, Astrovirus* | Azithromycin | Yes | Yes |
| ***37*** | **X** | **-** | *Salmonella* | 3GC | Yes | Yes |
| ***38*** | **-** | **X** | STEC, *C. jejuni* | Azithromycin | No | Yes |
| ***39*** | **-** | **X** | EIEC, STEC | Azithromycin | No | No |
| ***40*** | **-** | **X** | Shigella, ETEC | Azithromycin | No | Yes |
| ***41*** | **-** | **X** | *Salmonella* | Azithromycin | No | Yes |
| ***42*** | **-** | **X** | EIEC, *Norovirus*, *Giardia lamblia* | Metronidazole / Azithromycin | No | No |
| ***43*** | **-** | **X** | EIEC | Azithromycin | Yes | No |
| ***44*** | **-** | **X** | *Shigella* | Azithromycin | No | Yes |
| ***45*** | **-** | **X** | *C. jejuni, Salmonella*, *Cryptosporidium spp, Giardia lamblia*, *Sapovirus* | Metronidazole / Azithromycin | No | Yes |
| ***46*** | **-** | **X** | EIEC, *Pleisomonias shigelloides,* *Giardia lamblia* *Adenovirus* | Metronidazole | No | Yes |
| ***47*** | **-** | **X** | *C. difficile* | Azithromycin | Yes | No |
| ***48*** | **-** | **X** | *Salmonella* | 3GC | Yes | Yes |
| ***49*** | **-** | **X** | EIEC, *C. jejuni, Astrovirus* | Azithromycin | No | Yes |

^a^The appropriateness of antibiotic therapies was retrospectively assessed for the purpose of this study. EAEC, enteroaggregative *Escherichia coli*; STEC, Shiga toxin-producing *E. coli*; EPEC, enteropathogen *E. coli*; ETEC, enterotoxinogen *E. coli*; EIEC, enteroinvasive *E. coli*; 3GC, third-generation cephalosporins.
